# Clean Grinding Technique: A Facile Synthesis and In Silico Antiviral Activity of Hydrazones, Pyrazoles, and Pyrazines Bearing Thiazole Moiety against SARS-CoV-2 Main Protease (M^pro^)

**DOI:** 10.3390/molecules25194565

**Published:** 2020-10-06

**Authors:** Sraa Abu-Melha, Mastoura M. Edrees, Sayed M. Riyadh, Mohamad R. Abdelaziz, Abdo A. Elfiky, Sobhi M. Gomha

**Affiliations:** 1 Department of Chemistry, Faculty of Science, King Khalid University, Abha 61413, Saudi Arabia; sabomlha@kku.edu.sa; 2 Department of Organic Chemistry, National Organization for Drug Control and Research (NODCAR), Giza 12311, Egypt; 3 Department of Chemistry, Faculty of Science, Cairo University, Giza 12613, Egypt; riyadh1993@hotmail.com; 4 Department of Chemistry, Faculty of Science, Taibah University, Al-Madinah Al-Munawarah 30002, Saudi Arabia; 5 Department of Pharmaceutical Chemistry, Faculty of Pharmacy, MIU University, Cairo 19648, Egypt; sgomaa@sci.cu.edu.eg; 6 Biophysics Department, Faculty of Science, Cairo University, Giza 12613, Egypt; abdo@sci.cu.edu.eg; 7 Department of Chemistry, Faculty of Science, Islamic University, Al-Madinah Al-Munawarah 42351, Saudi Arabia

**Keywords:** hydrazide, hydrazone, condensation, cyclo-condensation, docking, SARS-CoV-2 M^pro^

## Abstract

A novel series of some hydrazones bearing thiazole moiety were generated via solvent-drop grinding of thiazole carbohydrazide **2** with various carbonyl compounds. Also, dehydrative-cyclocondensation of **2** with active methylene compounds or anhydrides gave the respective pyarzole or pyrazine derivatives. The structures of the newly synthesized compounds were established based on spectroscopic evidences and their alternative syntheses. Additionally, the anti-viral activity of all the products was tested against SARS-CoV-2 main protease (M^pro^) using molecular docking combined with molecular dynamics simulation (MDS). The average binding affinities of the compounds **3a**, **3b**, and **3c** (−8.1 ± 0.33 kcal/mol, −8.0 ± 0.35 kcal/mol, and −8.2 ± 0.21 kcal/mol, respectively) are better than that of the positive control Nelfinavir (−6.9 ± 0.51 kcal/mol). This shows the possibility of these three compounds to effectively bind to SARS-CoV-2 Mpro and hence, contradict the virus lifecycle.

## 1. Introduction

**Figure 1:**
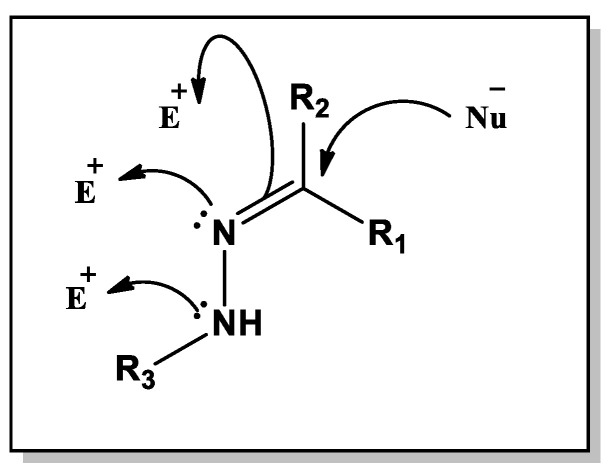
Structure and reactivity of hydrazones.

Clean chemistry is a multidisciplinary concept for the judicious use of chemical reactions and prevents its pollution. It involves the designing of chemical products by using environmentally safe procedures that reduce the generation of hazardous substances, as well as the cost [1]. Grinding methods are appealing eco-friendly techniques for efficient organic synthesis with many advantages, such as: mild reaction conditions, easy separation and purifications [2,3], high efficiency and selectivity [4], and environmental acceptability [5]. In the meantime, hydrazone is an effective nucleus because hydrazine-containing compounds (R_1_R_2_C=NNH-R_3_) offer a wide range of biological activities, including anticholinesterase [6], antimycobacterial [7], anti-cancer [8], antibacterial, anti-inflammatory, anti-viral, antifungal, anti-tubercular, analgesic, muscle relaxants, and antihistamines [9,10,11,12]. Hydrazones possessing an azometine -NHN=CH- proton constitute an important class of compounds for new drug development. Both nitrogen atoms of the hydrazone group are nucleophilic and the carbon atom of the hydrazone group has both electrophilic and nucleophilic properties (Figure 1). These structural fragments are principally responsible for the physical and chemical properties of hydrazones. Therefore, many researchers have synthesized these compounds as target structures and evaluated their biological activities. These observations have been guiding the development of new hydrazones that possess varied biological activities [13,14,15].

Likewise, pyrazoline derivatives widely occur in nature in the form of alkaloids, vitamins, animal cells, and pigments. The unique structural array and pronounced pharmacological activities [16,17] of pyrazolines have made them attractive synthetic targets for many researchers. In continuation of our interest in green synthesis of heterocycles [2,18,19,20,21,22,23], herein, we propose an easy, efficient, solid-state grinding method for the preparation of hydrazones. This method can also be extended to the reactions of hyrazide with active methylene and anhydride compounds. 

SARS-CoV-2 represents the century challenge to human beings. It represents the primary agent for the novel, deadly, pandemic of COVID-19 worldwide [24]. The main viral protease (M^pro^) of SARS-CoV-2 sheds drug designers due to its vital role in keeping the virus alive [25]. M^pro^ is a crucial enzyme for the viral polypeptide processing into the different enzymes that maintain the life cycle of the SARS-CoV-2. Hence, blocking M^pro^ activity is vital in combating COVID-19. Different compounds (natural and synthetic) are used as possible potential therapeutics against SARS-CoV-2 M^pro^. In this study, we tested some hydrazones, pyarzoles, and pyrazines derivatives synthesized using a clean grinding technique.

Molecular modeling represents an excellent method used to judge the chemical reactivity in silico [26]. The fast improvement in the last two decades makes molecular modeling the second hand for drug designers. It helps reduce time, money, and effort in the journey of drug design [27]. It was successful in helping find potent anti-viral agents against many viruses, like Human Immunodeficiency Virus (HIV) and Hepatitis C Virus (HCV) [28,29,30]. Additionally, computer-aided drug design is used to find the potency of drugs and natural products against SARS-CoV-2 proteins during the last few months [31,32,33,34]. In this study, molecular docking combined with molecular dynamics simulation is used to test the different compounds against SARS-CoV-2 M^pro^.

## 2. Results and Discussion

### 2.1. Chemistry

**Scheme 1:**
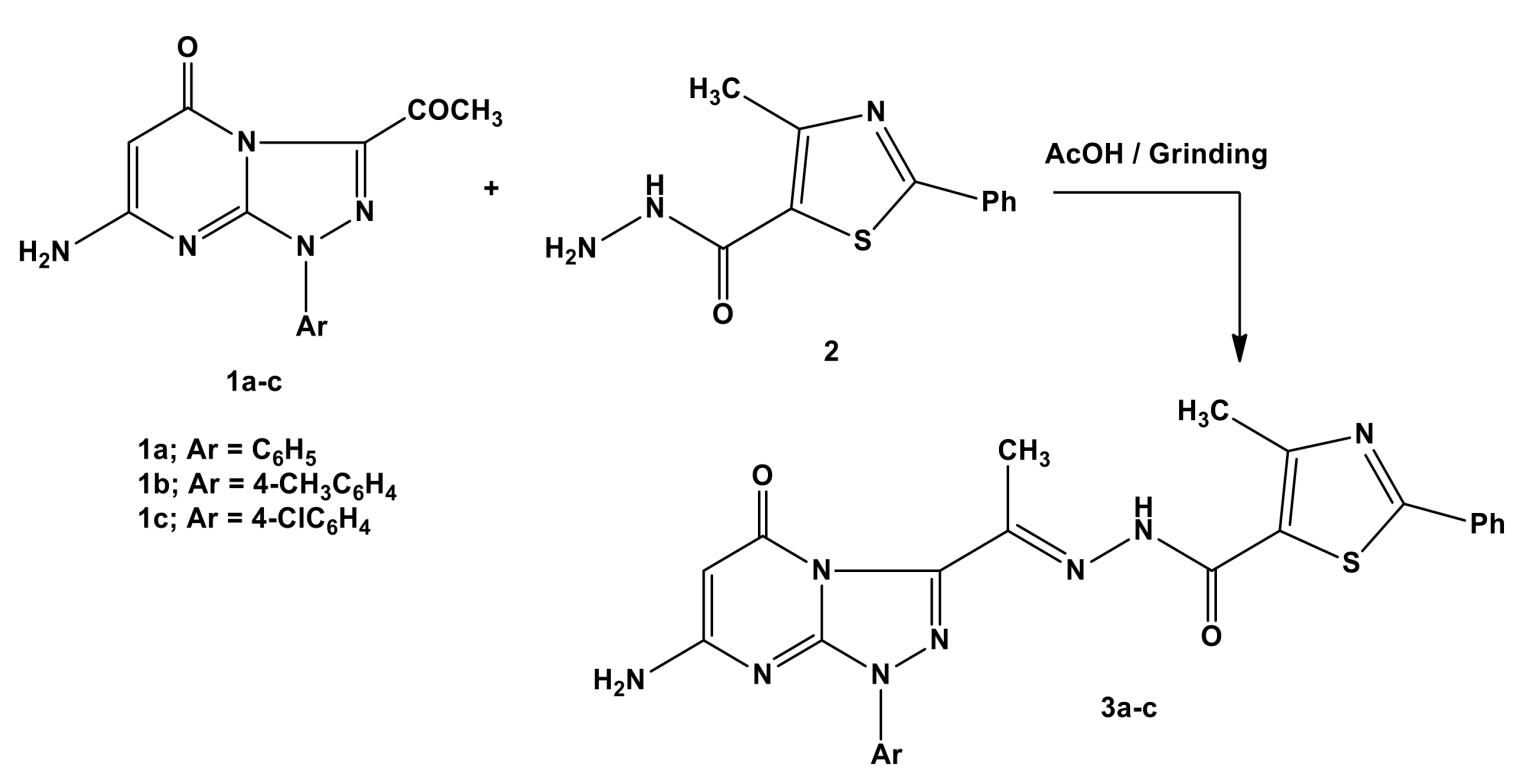
Synthesis of triazolopyrimidine derivatives **3a**–**c**.

An initial endeavor was to investigate the condensation reactions under solid-state conditions. Thus, grinding of 3-acetyl-7-amino-1-aryl-[1,2,4]triazolo [4,3-*a*]pyrimidin-5(1*H*)-one (**1**) [35] and 4-methyl-2-phenylthiazole -5-carbohydrazide (**2**) [36], in the presence of a few drops of acetic acid at room temperature, gave the condensation products *N*’-[1-(7-amino-5-oxo-1-aryl-1,5-dihydro-[1,2,4] triazolo[4,3-*a*]pyrimidin-3-yl)ethylidene]-4-methyl-2-phenylthiazole-5-carbohydrazides (**3a**–**c**) (Scheme 1). 

The spectral data are in accordance with the isolated products **3a**–**c** (see the Supplementary Figure S1). ^1^H-NMR revealed two singlet signals at *δ* = (2.37–2.45) and (2.58–2.64) ppm due to (CH_3_-C=N) and (CH_3_-thiazole) [34], respectively. Another three singlet signals at *δ* = (5.30–5.36), (6.92–6.98), and (9.42–9.59) ppm were revealed, attributed to (CH-pyrimidine), (NH_2_) [32], and (NH-CO) [34], respectively. The molecular ion peaks and microanalysis results for the synthesized products were also in accordance with the theoretical calculation. 

**Scheme 2:**
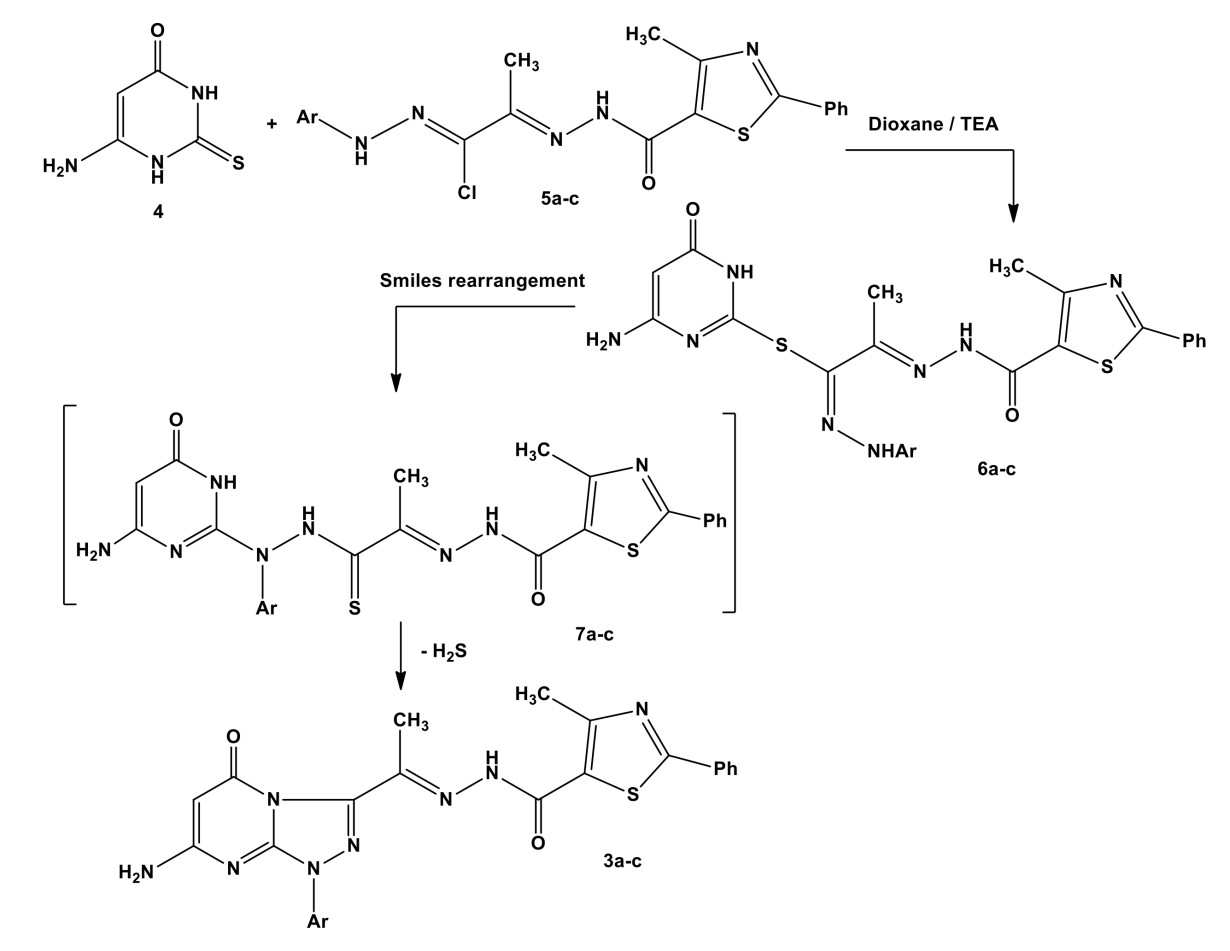
Alternative synthesis of triazolopyrimidine derivatives **3a**–**c**.

To verify the assigned structure of the products **3a**–**c**, we have synthesized these compounds by alternative methods. Thus, treatment of aminothiouracil (**4**) with 2-[2-(4-methyl-2- phenylthiazole-5-carbonyl) hydrazono]-*N*-arylpropanehydrazonoyl chlorides (**5a**–**c**) [37] in dioxane, in the presence of a catalytic amount of triethylamine, under thermal conditions, gave the authentic products identical in all respects (mp, mixed mp, IR) with the isolated products **3a**–**c** via Smiles rearrangement [38] and elimination of H_2_S (Scheme 2).

**Scheme 3:**
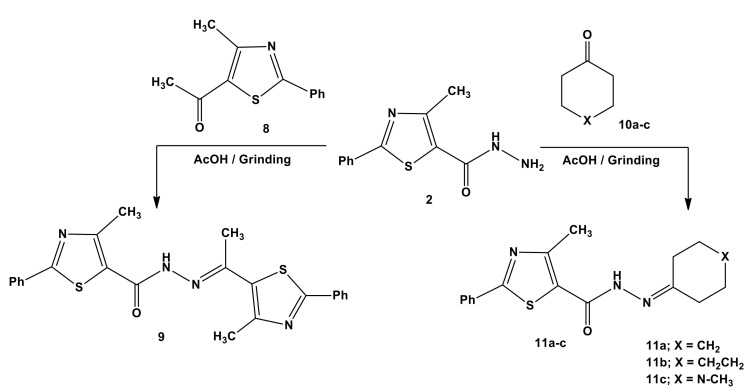
Synthesis of thiazoles **9** and **11a**–**c**.

Next, we investigated the derivatization of acylhydrazide group with thiazole moiety to generate the novel hydrazone templets. Thus, the reaction of 4-methyl-2-phenylthiazole- 5-carbohydrazide (**2**) with 2-phenyl-4-methyl-5-acetylthiazole (**8**) [39] or cyclic ketones **10a**–**c** under solid-state conditions furnished the corresponding acylhydrazones **9** or **11a**–**c**, respectively (Scheme 3). Microanalyses and spectral data (IR, ^1^H-NMR, ^13^C-NMR, and MS) are compatible with the structures of hydazones (see the Supplementary Figure S2). 

**Scheme 4:**
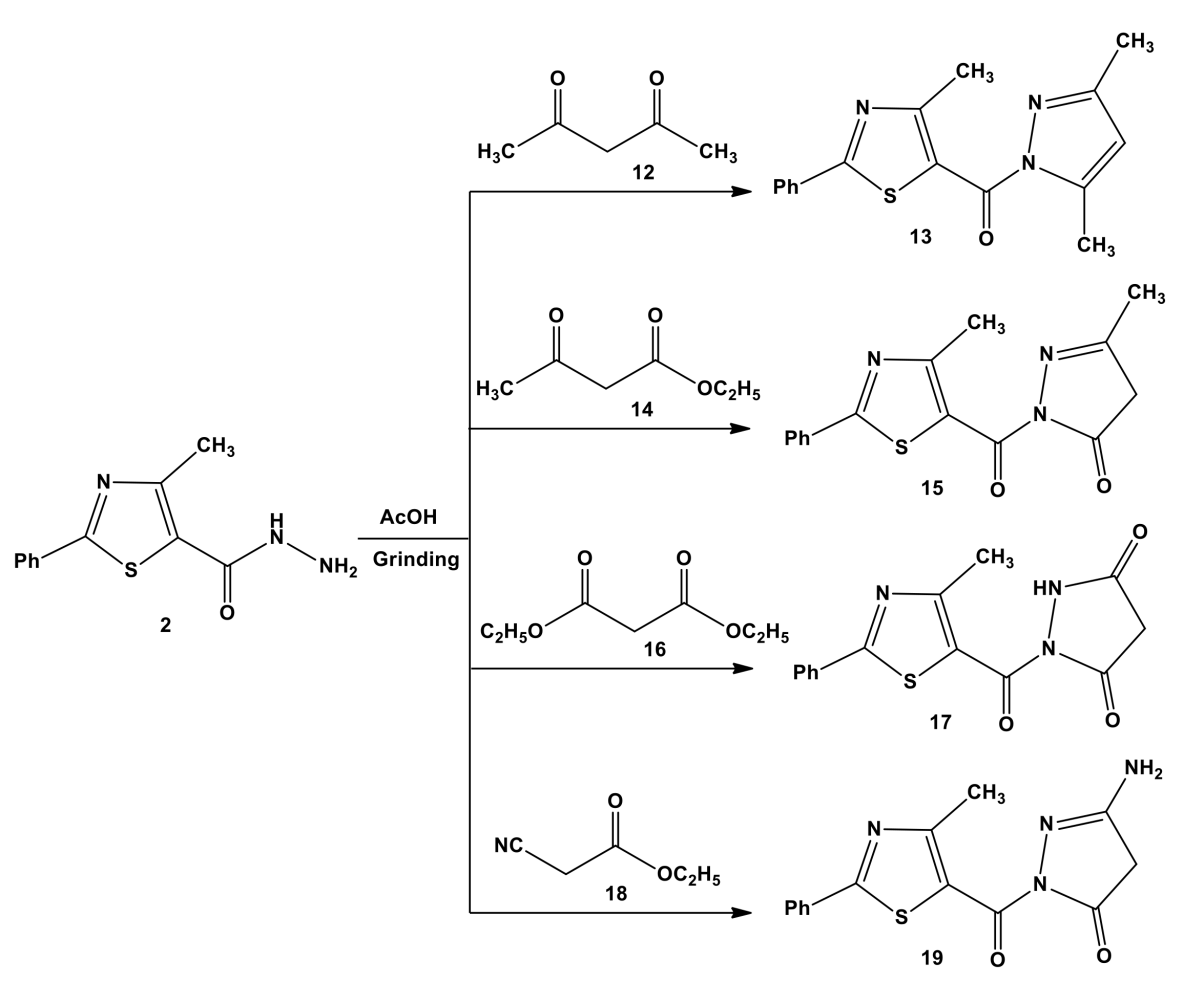
Synthesis of pyrazoles **13**, **15**, **17**, and **19**.

To generalize the grinding technique, the cyclo-condensation reactions were examined. As a consequence, treatment of acid hydrazide **2** with active methylene compounds, such as 2,4-pentanedione (**12**), ethyl 3-oxobutanoate (**14**), diethylmalonate (**16**), and ethyl cyanoacetate (**18**), under grinding conditions led to the formation of (3,5-dimethyl-1*H*-pyrazol-1-yl)(4-methyl- 2-phenylthiazol-5-yl)methanone (**13**), (3-methyl-5-oxo-1,4-dihydro-pyrazol-1- yl)(4-methyl-2-phenyl thiazol-5-yl)methanone (**15**), 1-(4-methyl-2-phenylthiazole-5-carbonyl) pyrazolidine-3,5-dione (**17**), and 3-amino-1-(4-methyl-2-phenylthiazole-5- carbonyl)-1,4-dihydropyrazol-5-one (**19**), respectively (Scheme 4). ^1^H-NMR spectrum of compound **13** showed that the characteristic singlet at *δ* = 6.05 ppm corresponds to pyrazole-CH [40]. Also, the characteristic (CH_2_) protons of pyrazole ring appeared as a singlet at *δ* = 5.25 ppm [41] to confirm the structure of compound **15**. Furthermore, the presence of a singlet at *δ* = 5.94 ppm corresponding to the (NH_2_) protons proved the formation of the compound **19**. In the IR spectra, all pyrazole and pyrazoline compounds displayed a characteristic band near 1690 cm^−1^ attributed to C=O bridge stretching vibration (see the Supplementary Figure S3).

**Scheme 5:**
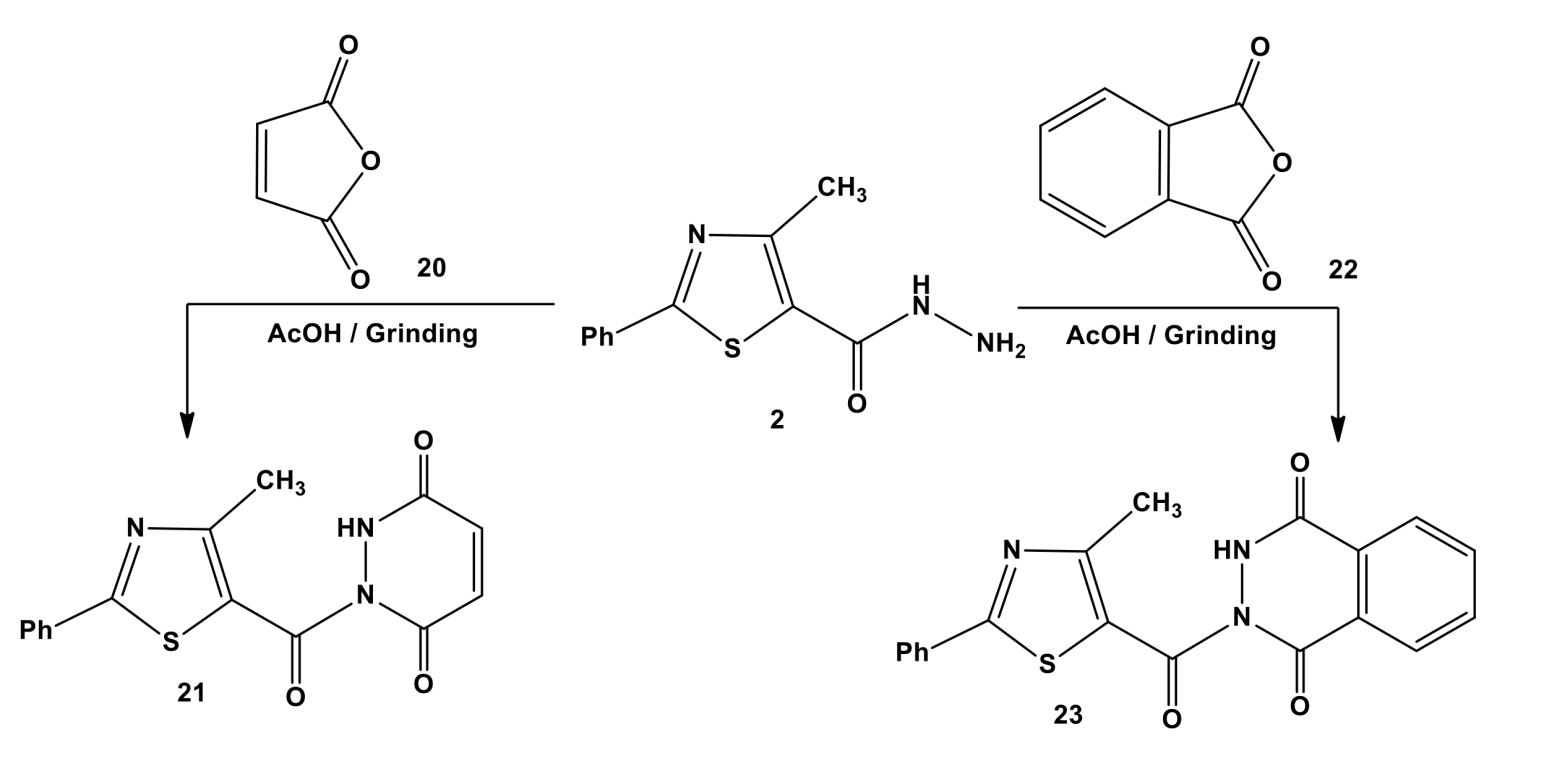
Synthesis of pyridazines **21** and **23**.

Additionally, the reaction of acid hydrazide **2** with maleic anhydride (**20**) or phthalic anhydride (**22**) resulted in ring-opening and ring closure to give the corresponding 1-(4-methyl-2-phenylthiazole-5-carbonyl)-1,2-dihydropyridazine-3,6-dione (**21**) or 2-(4-methyl-2-phenyl-thiazole-5-carbonyl)-2,3-dihydro phthalazine-1,4-dione (**23**), respectively (Scheme 5). Spectral data (IR, ^1^H-NMR, ^13^C-NMR, and MS) and elemental analyses are in accordance with the structures of the isolated products (see the Supplementary Figure S4).

### 2.2. Molecular Simulations

**Figure 2:**
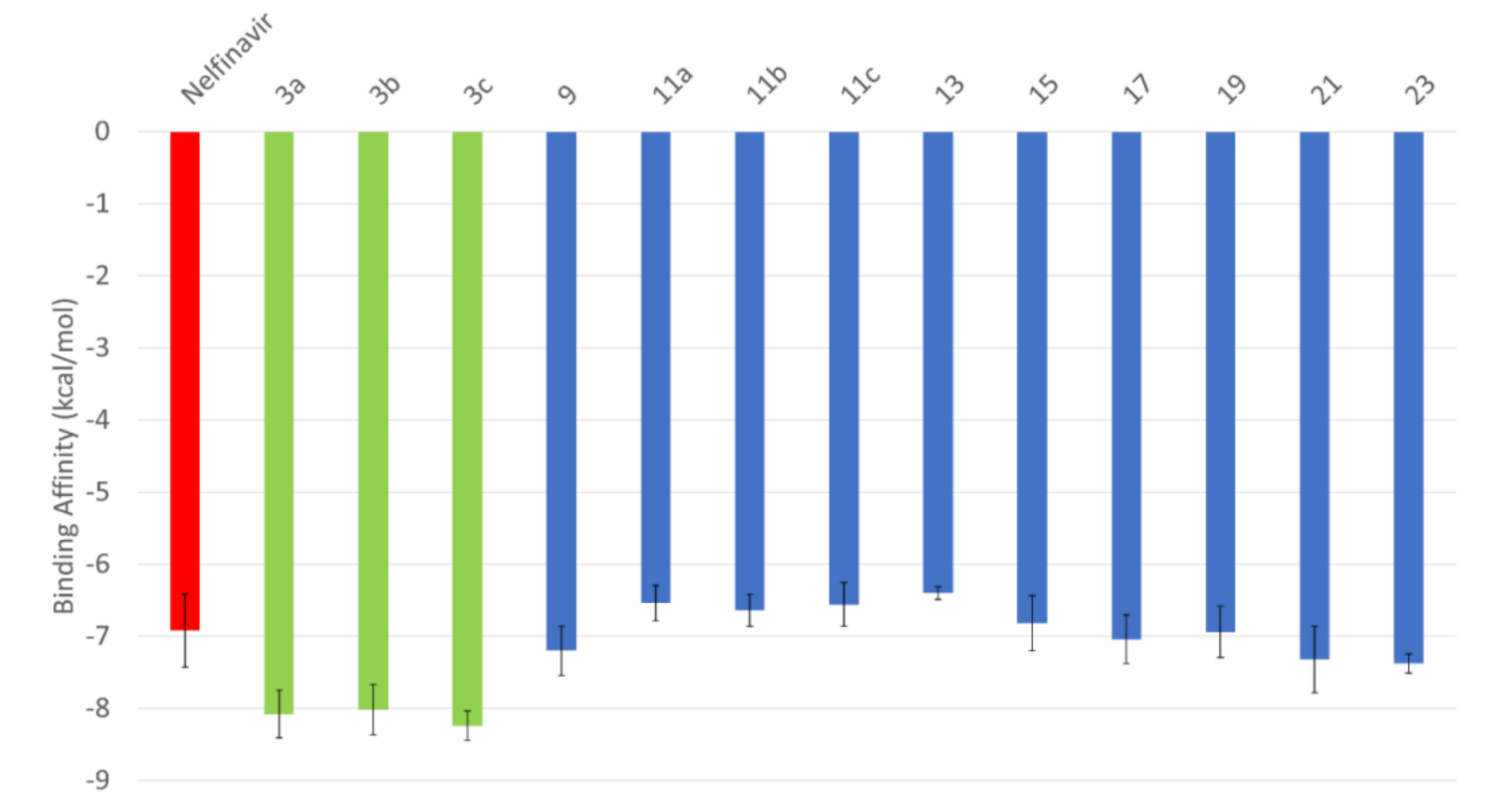
Average binding energies (in kcal/mol) of the compounds of Nelfinavir (red) **3a**, **3b**, and **3c** (green), and **9**, **11a**, **11b**, **11c**, **13**, **15**, **17**, **19**, **21**, and **23** (blue) docked into the active site residues of SARS-CoV-2 PL^pro^. Error bars represent the standard deviations.

The structures are tested for their anti-viral activity against SARS-CoV-2 main protease (M^pro^) using molecular docking combined with molecular dynamics simulation (MDS) for up to 100 nanoseconds (see the Supplementary Figures S5 and S6). Figure 2 shows the average binding affinities of the compounds calculated using AutoDock Vina [42]. The anti-viral Nelfinavir (red column) is tested as a positive control. The error bars represent the standard deviation (SD) from all the docking trials using the different conformations of SARS-CoV-2 M^pro^. As reflected from Figure 2, the average binding affinities of the compounds **3a**, **3b**, and **3c** (green columns) (−8.1 ± 0.33 kcal/mol, −8.0 ± 0.35 kcal/mol, and −8.2 ± 0.21 kcal/mol, respectively) are better (less) than that of the positive control Nelfinavir (−6.9 ± 0.51 kcal/mol). This shows the possibility of these three compounds to effectively bind to SARS-CoV-2 M^pro^ and hence, contradict the virus lifecycle. Additionally, the compounds **9**, **11a**, **11b**, **11c**, **13**, **15**, **17**, **19**, **21**, and **23** have average binding affinities comparable to Nelfinavir (−6.4 ± 0.09 kcal/mol for compound **13** down to −7.4 ± 0.13 kcal/mol for compound **23**); hence, it may bind to SARS-CoV-2 M^pro^ and stop the viral infection as well. 

**Figure 3:**
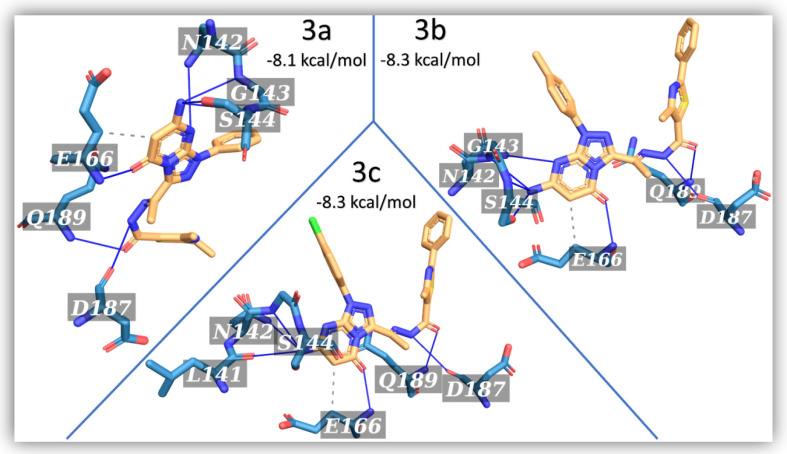
The interaction pattern (after docking) for the best three thiazole compounds, **3a**, **3b**, and **3c**, depicted using PyMOL software. The compounds are shown in yellow sticks, while the interacting residues form SARS-CoV-2 M^pro^ are depicted in blue sticks labeled with its one-letter codes. The formed H-bonds and hydrophobic contacts are shown in blue and dashed-gray lines, respectively.

**Figure 4:**
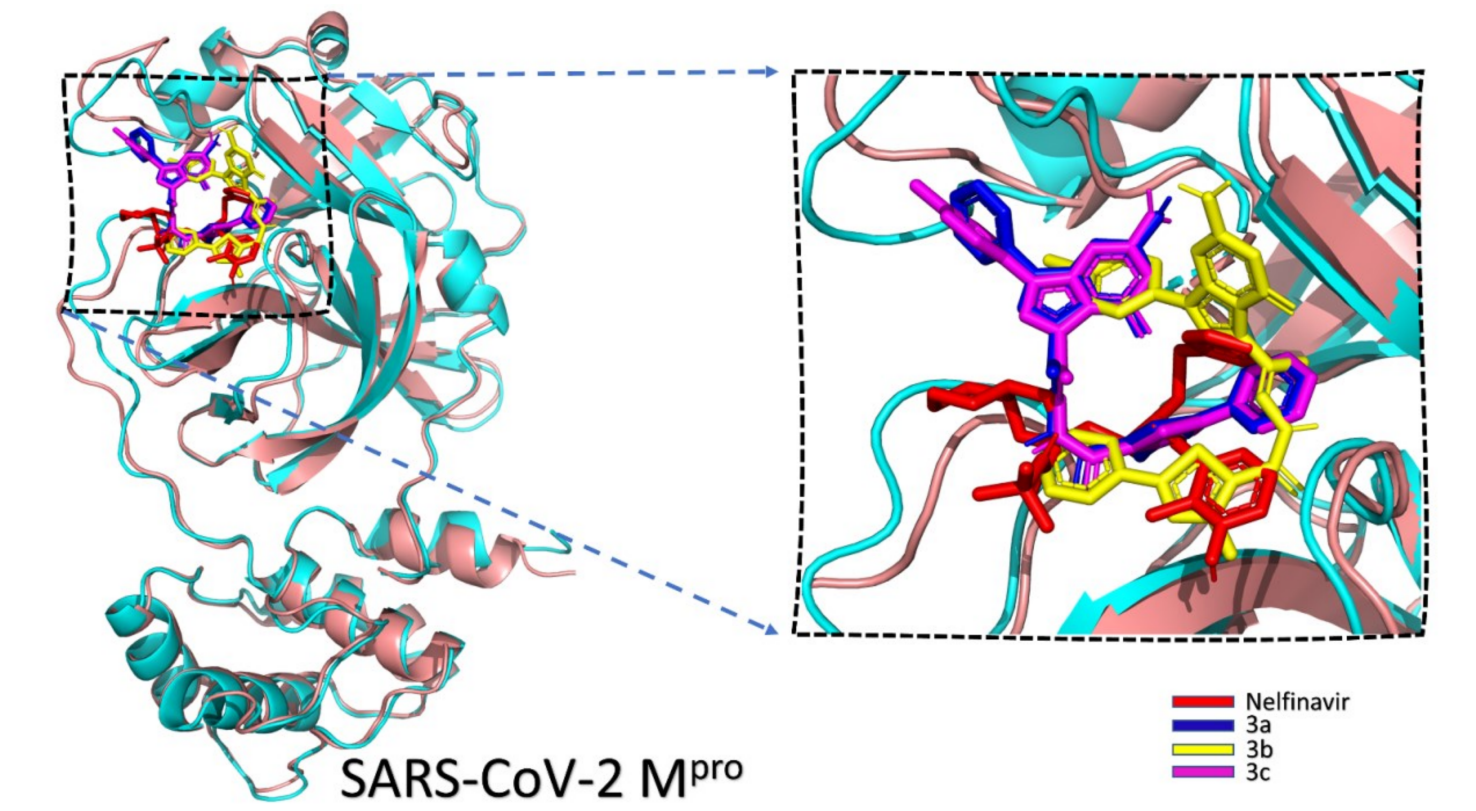
The superposition of the positive control (Nelfinavir) and the best three compounds, **3a**, **3b**, and **3c**, depicted using PyMOL software. The compounds are shown in colored sticks, and M^pro^ in colored cartoons.

To further analyze the binding mode, the docking complexes are analyzed by the aid of the Protein–Ligand Interaction Profiler (PLIP) software [43]. Table 1 shows the interaction profiles of the compounds **3a**, **3b**, **3c**, **9**, **11a**, **11b**, **11c**, **13**, **15**, **17**, **19**, **21**, and **23** docked into the active site residues of SARS-CoV-2 PL^pro^. The most reported types of interaction were the H-bonds and hydrophobic contacts. Noticeably, the most reported interacting residue in almost all complexes is E166. For the best three compounds (**3a**, **3b**, and **3c**), the interaction is almost identical with 7 H-bonds (N142, G143, S144, E166, D187, and Q189) and one hydrophobic contact (E166). These three compounds have the lowest binding energy values (–8.1 to –8.3 kcal/mol). For the other compounds, the number of formed interactions is fewer. This is reflected in the binding energy values (–6.4 to –7.3 kcal/mol). The interaction pattern for the best three thiazole compounds, **3a**, **3b**, and **3c**, are depicted in Figure 3. These three compounds can interact with the same binding site pocket of the SARS-CoV-2 M^pro^ as reflected in the superposition (Figure 4) of the compounds after docking. The compounds **3a**, **3b**, and **3c** proved their ability to bind firmly to the active site of the protein, and hence could be successful candidates against COVID-19. Experimental validation of the binding potency of the three compounds against SARS-CoV-2 M^pro^ is suggested as a future recommendation. Further in-depth MDS analysis of the best three compounds is suggested as future work. Additionally, we suggest testing these compounds against other enzymes for SARS-CoV-2, such as the papain-like protease (PL^pro^) and RNA-dependent RNA polymerase (RdRp). It may bind firmly to these targets and may help inactivate SARS-CoV-2. 

## 3. Experimental

### 3.1. Chemistry

An electrothermal Gallenkamp apparatus was operated to measure the melting points for the newly synthesized compounds (Bibby Sci. Lim. Stone, Staffordshire, UK). Pye-Unicam SP300 instrument in potassium bromide discs was used to measure Infra-Red, IR spectra (Shimadzu, Tokyo, Japan). A Varian Mercury VXR-300 spectrometer (300 MHz for ^1^H-NMR and 75 MHz for ^13^C-NMR) (Varian, Inc., Karlsruhe, Germany) was manipulated to measure the ^1^H NMR, and ^13^C-NMR spectra and the chemical shifts were related to that of the solvent. GCMS-Q1000-EX Shimadzu spectrometers (Tokyo, Japan) were conducted to record the mass spectra of the samples on the ionizing voltage at 70 eV. Elemental analyses were carried out at the Microanalytical Centre of Cairo University, Giza, Egypt.

#### General Procedure for the Synthesis of Compounds **3a**–**c**, **9**, **11a**–**c**, **16**–**19**, **22**, and **23**

A mixture of 4-methyl-2-phenylthiazole-5-carbohydrazide (**2**) (0.233 g, 1 mmol) and appropriate carbonyl compounds (1 mmol) was ground in a mortar at room temperature in the presence of a few drops of acetic acid for 10–20 min (monitored through Thin Layer Chromatography, TLC). The reaction mixture was poured into water, filtered, and washed with water and ethanol. The crude products were recrystallized from the appropriate solvent. 

Alternative Synthesis of Compounds **3a**–**c**:

Equimolecular amounts of aminothiouracil (**4**) (0.143 g, 1 mmol) and 2-[2-(4-methyl-2-phenyl thiazole-5-carbonyl)hydrazono]-*N*-arylpropanehydrazonoyl chlorides (**5a**–**c**) (1 mmol) were dissolved in dioxane (15 mL), containing a few drops of triethylamine. The reaction mixture was refluxed for 2 h and poured into ice/HCl. The precipitate formed was collected by filtration, dried, and recrystallized from appropriate solvent to give the respective products identical in all respects (mp and mixed mp) with **3a**–**c**.

*N′-[1-(7-Amino-5-oxo-1-phenyl-1,5-dihydro-[1,2,4]triazolo[4,3-a]pyrimidin-3-yl)ethylidene]-4-methyl-2-phenylthiazole-5-carbohydrazide* (**3a**). Brown powder (88%); mp = 168–170 °C (EtOH); IR (KBr) *v* = 3485, 3300 (NH_2_), 3224 (NH), 1683 (C=O), 1601 (C=N) cm^−1^; ^1^H-NMR (DMSO-*d_6_*, 300 MHz): *δ* = 2.37 (3H, s, CH_3_–C=N–NH), 2.58 (3H, s, thiazole–CH_3_), 5.32 (1H, s, pyrimidine-CH), 6.92 (2H, s, NH_2_), 7.22-7.39 (10H, m, Ar–H), 9.42 (1H, s, NH); ^13^C-NMR (DMSO-*d_6_*, 75 MHz): *δ* = 13.8 (CH_3_-C=N-NH), 15.2 (CH_3_-thiazole), 81.8, 121.4, 125.6, 127.8, 128.3, 128.4, 129.0, 129.2, 129.6, 138.6, 142.8, 143.4, 147.8, 158.0, 159.8, 163.7, 165.7, 171.5; EIMS *m*/*z* (%): 484 [M^+^] (51), 461 (37), 272 (70), 180 (59), 59 (100). Anal. calcd for C_24_H_20_N_8_O_2_S (484.14): C, 59.49; H, 4.16; N, 23.13; S, 6.62. Found: C, 59.66; H, 4.28; N, 23.32; S, 6.65.

*N′-[1-(7-Amino-5-oxo-1-(4-methylphenyl)-1,5-dihydro-[1,2,4]triazolo[4,3-a]pyrimidin-3-yl)ethylidene]-4-methyl-2-phenylthiazole-5-carbohydrazide* (**3b**). Brown powder (89%); mp = 148–150 °C (EtOH); IR (KBr) *v* = 3477, 3310 (NH_2_), 3220 (NH), 1687 (C=O), 1602 (C=N) cm^−1^; ^1^H-NMR (DMSO-*d_6_*, 300 MHz): *δ* = 2.23 (3H, s, Ar-CH_3_), 2.42 (3H, s, CH_3_–C=N–NH), 2.61 (3H, s, thiazole–CH_3_), 5.30 (1H, s, pyrimidine-CH), 6.93 (2H, s, NH_2_), 7.39-8.05 (9H, m, Ar–H), 9.50 (1H, s, NH); ^13^C-NMR (DMSO-*d_6_*, 75 MHz): *δ* = 15.2 (CH_3_-C=N-NH), 17.8 (CH_3_-thiazole), 21.2 (Ar-CH_3_), 81.1, 122.9, 126.8, 127.9, 128.1, 130.5, 131.9, 132.2, 135.4, 138.9, 140.8, 142.1, 145.5, 148.1, 153.3, 158.9, 162.8, 169.6; EIMS *m*/*z* (%): 498 [M^+^] (30), 472 (60), 407 (80), 64 (100). Anal. calcd for C_25_H_22_N_8_O_2_S (498.16): C, 60.23; H, 4.45; N, 22.48; S, 6.43. Found: C, 60.44; H, 4.38; N, 22.32; S, 6.52.

*N′-[1-(7-Amino-5-oxo-1-(4-chlorophenyl)-1,5-dihydro-[1,2,4]triazolo[4,3-a]pyrimidin-3-yl)ethylidene]-4-methyl-2-phenylthiazole-5-carbohydrazide* (**3c**). Brown powder (92%); mp = 155–157 °C (EtOH); IR (KBr) *v* = 3440, 3340 (NH_2_), 3235 (NH), 1689 (C=O), 1602 (C=N) cm^−1^; ^1^H-NMR (DMSO-*d_6_*, 300 MHz): *δ* = 2.45 (3H, s, CH_3_–C=N–NH), 2.64 (3H, s, thiazole–CH_3_), 5.36 (1H, s, pyrimidine-CH), 6.98 (2H, s, NH_2_), 7.41-8.26 (9H, m, Ar–H), 9.59 (1H, s, NH); ^13^C-NMR (DMSO-*d_6_*, 75 MHz): *δ* = 16.4 (CH_3_-C=N-NH), 18.1 (CH_3_-thiazole), 82.1, 121.9, 123.1, 126.8, 127.9, 128.8, 130.6, 131.3, 132.8, 136.4, 137.8, 141.9, 142.8, 145.5, 153.3, 159.6, 162.8, 169.9; EIMS *m*/*z* (%): 520 [M^+^ + 2] (6), 518 [M^+^] (15), 456 (41), 272 (46), 200 (100). Anal. calcd for C_24_H_19_ClN_8_O_2_S (518.10): C, 55.54; H, 3.69; N, 21.59; S, 6.18. Found: C, 55.46; H, 3.71; N, 21.42; S, 6.23.

*N′-[1-(4-Methyl-2-phenylthiazol-5-yl)ethylidene]-2-phenyl-4-methylthiazole-5-carbohydrazide* (**9**). Yellow powder (94%); mp = 242–244 °C (Dioxane); IR (KBr) *v* = 3158 (NH), 1668 (C=O) cm^−1^; ^1^H-NMR (DMSO-*d_6_*, 300 MHz): *δ* = 2.44 (3H, s, CH_3_–C=N–NH), 2.63 (3H, s, thiazole–CH_3_), 2.75 (3H, s, thiazole–CH_3_), 7.51-8.01 (10H, m, Ar–H), 11.01 (1H, s, NH); ^13^C-NMR (DMSO-*d_6_*, 75 MHz): *δ* = 13.5 (CH_3_-C=N-NH), 14.0 (CH_3_-thiazole), 16.2 (CH_3_-thiazole), 125.5, 125.6, 127.8, 128.3, 128.6, 128.8, 128.9, 129.5, 129.6, 130.9, 138.5, 142.5, 144.1, 148.2, 164.7, 170.3; EIMS *m*/*z* (%): 432 [M^+^] (70), 202 (100), 71 (30). Anal. calcd for C_23_H_20_N_4_OS_2_ (432.11): C, 63.86; H, 4.66; N, 12.95; S, 14.82. Found: C, 63.65; H, 4.48; N, 13.02; S, 14.95.

*N′-Cyclohexylidene-4-methyl-2-phenylthiazole-5-carbohydrazide* (**11a**). Yellowish-white powder (88%); mp = 186–188 °C (EtOH); IR (KBr) *v* = 3187 (NH), 1675 (C=O) cm^−1^; ^1^H-NMR (DMSO-*d_6_*, 300 MHz): *δ* = 1.92 (6H, m, 3 CH_2_), 2.49 (4H, m, 2 CH_2_), 2.64 (3H, s, thiazole–CH_3_), 7.52-7.96 (5H, m, Ar–H), 10.12 (1H, s, NH); ^13^C-NMR (DMSO-*d_6_*, 75 MHz): *δ* = 18.2 (CH_3_-thiazole), 24.9 (CH_2_), 27.8 (CH_2_), 34.9 (CH_2_), 125.3, 128.7, 130.8, 132.4, 142.3, 143.8, 144.5, 158.3, 168.3; EIMS *m*/*z* (%): 313 [M^+^] (10), 257 (100), 80 (90). Anal. calcd for C_17_H_19_N_3_OS (313.12): C, 65.15; H, 6.11; N, 13.41; S, 10.23. Found: C, 65.25; H, 6.18; N, 13.32; S, 10.35.

*N′-Cycloheptylidene-4-methyl-2-phenylthiazole-5-carbohydrazide* (**11b**). Yellowish-white powder (87%); mp = 211–213 °C (Dioxane); IR (KBr) *v* = 3157 (NH), 1675 (C=O) cm^−1^; ^1^H-NMR (DMSO-*d_6_*, 300 MHz): *δ* = 1.62 (4H, m, 2 CH_2_), 1.89 (4H, m, 2 CH_2_), 2.49 (4H, m, 2 CH_2_), 2.65 (3H, s, thiazole–CH_3_), 7.53-7.98 (5H, m, Ar–H), 10.18 (1H, s, NH); ^13^C-NMR (DMSO-*d_6_*, 75 MHz): *δ* = 18.2 (CH_3_-thiazole), 25.9 (CH_2_), 27.8 (CH_2_), 35.9 (CH_2_), 124.8, 128.9, 130.5, 132.4, 142.3, 143.5, 144.4, 158.3, 161.9, 168.8; EIMS *m*/*z* (%): 327 [M^+^] (60), 202 (100), 71 (40). Anal. calcd for C_18_H_21_N_3_OS (327.14): C, 66.03; H, 6.46; N, 12.83; S, 9.79. Found: C, 65.95; H, 6.48; N, 13.02; S, 9.85.

*N′-(1-Methylpiperidin-4-ylidene)-2-phenyl-4-methyl-thiazole-5-carbohydrazide* (**11c**). White powder (84%); mp = 172–174 °C (EtOH/dioxane); IR (KBr) *v* = 3188 (NH), 1681 (C=O) cm^−1^; ^1^H-NMR (DMSO-*d_6_*, 300 MHz): *δ* = 1.91 (4H, m, 2 CH_2_), 2.49 (4H, m, 2 CH_2_), 2.66 (3H, s, thiazole–CH_3_), 3.31 (3H, s, N-CH_3_), 7.52-7.97 (5H, m, Ar–H), 9.93 (1H, s, NH); ^13^C-NMR (DMSO-*d_6_*, 75 MHz): *δ* = 18.6 (CH_3_-thiazole), 24.9 (CH_2_), 47.8 (N-CH_3_), 54.9 (CH_2_), 123.9, 127.8, 131.8, 133.4, 142.3, 143.8, 146.5, 160.3, 168.8; EIMS *m*/*z* (%): 328 [M^+^] (30), 257 (100), 154 (80), 80 (50). Anal. calcd for C_17_H_20_N_4_OS (328.14): C, 62.17; H, 6.14; N, 17.06; S, 9.76. Found: C, 62.25; H, 6.11; N, 17.12; S, 9.55.

*(3,5-Dimethyl-1H-pyrazol-1-yl)(4-methyl-2-phenylthiazol-5-yl)methanone* (**13**). Yellow powder (87%); mp = 200–202 °C (Dioxane); IR (KBr) *v* = 1685 (C=O) cm^−1^; ^1^H-NMR (DMSO-*d_6_*, 300 MHz): *δ* = 2.18 (3H, s, pyrazole-CH_3_), 2.38 (3H, s, pyrazole-CH_3_), 2.64 (3H, s, thiazole–CH_3_), 6.05 (1H, s, pyrazole-CH), 7.52-8.02 (5H, m, Ar–H); ^13^C-NMR (DMSO-*d_6_*, 75 MHz): *δ* = 14.8 (CH_3_-pyrazole), 15.2 (CH_3_-pyrazole), 18.4 (CH_3_-thiazole), 127.8, 128.3, 130.8, 132.4, 142.3, 143.8, 146.5, 148.9, 150.3, 160.8, 168.8. EIMS *m*/*z* (%): 297 [M^+^] (10), 257 (30), 202 (100), 71 (60). Anal. calcd for C_16_H_15_N_3_OS (297.09): C, 64.62; H, 5.08; N, 14.13; S, 10.78. Found: C, 64.55; H, 5.11; N, 14.18; S, 10.58.

*(3-Methyl-5-oxo-1,4-dihydro-pyrazol-1-yl)(4-methyl-2-phenylthiazol-5-yl)methanone* (**15**). Brown powder (89%); mp = 136–138 °C (EtOH); IR (KBr) *v* = 1685 (2C=O) cm^−1^; ^1^H-NMR (DMSO-*d_6_*, 300 MHz): *δ* = 2.64 (3H, s, pyrazole-CH_3_), 2.68 (3H, s, thiazole–CH_3_), 5.25 (2H, s, pyrazole-CH_2_), 7.53-7.98 (5H, m, Ar–H); ^13^C-NMR (DMSO-*d_6_*, 75 MHz): *δ* = 15.8 (CH_3_-pyrazole), 18.4 (CH_3_-thiazole), 46.2 (CH_2_-pyrazole), 125.8, 127.3, 130.8, 132.4, 141.9, 143.2, 146.4, 148.9, 162.8, 169.8. EIMS *m*/*z* (%): 299 [M^+^] (40), 257 (30), 202 (100), 71 (60). Anal. calcd for C_15_H_13_N_3_O_2_S (299.07): C, 60.19; H, 4.38; N, 14.04; S, 10.71. Found: C, 60.25; H, 4.21; N, 14.19; S, 10.88.

*1-(4-Methyl-2-phenylthiazole-5-carbonyl)pyrazolidine-3,5-dione* (**17**). Yellowish-white powder (86%); mp = 306–308 °C (DMF); IR (KBr) *v* = 3208 (NH), 1691, 1680 (3C=O) cm^−1^; ^1^H-NMR (DMSO-*d_6_*, 300 MHz): *δ* = 2.68 (3H, s, thiazole–CH_3_), 3.42 (2H, s, pyrazole-CH_2_), 7.53-7.99 (5H, m, Ar–H), 10.96 (1H, s, NH); ^13^C-NMR (DMSO-*d_6_*, 75 MHz): *δ* = 18.9 (CH_3_-thiazole), 46.2 (CH_2_-pyrazole), 125.8, 127.8, 131.1, 132.6, 141.9, 146.9, 148.9, 162.8, 165.6, 169.8. EIMS *m*/*z* (%): 301 [M^+^] (60), 257 (60), 202 (80), 80 (100). Anal. calcd for C_14_H_11_N_3_O_3_S (301.05): C, 55.81; H, 3.68; N, 13.95; S, 10.64. Found: C, 55.65; H, 3.41; N, 14.09; S, 10.83.

*3-Amino-1-(4-methyl-2-phenylthiazole-5-carbonyl)-1,4-dihydropyrazol-5-one* (**19**). White powder (85%); mp = 175–177 °C; IR (KBr) *v* = 3268, 3166 (NH_2_), 1683 (2C=O) cm^−1^; ^1^H-NMR (DMSO-*d_6_*, 300 MHz): *δ* = 2.66 (3H, s, thiazole–CH_3_), 3.45 (2H, s, pyrazole-CH_2_), 5.94 (2H, s, NH_2_), 7.55-8.01 (5H, m, Ar–H); ^13^C-NMR (DMSO-*d_6_*, 75 MHz): *δ* = 17.9 (CH_3_-thiazole), 51.2 (CH_2_-pyrazole), 124.8, 128.8, 130.1, 134.6, 141.9, 145.9, 148.3, 160.7, 165.6, 168.8. EIMS *m*/*z* (%): 300 [M^+^] (70), 275 (30), 202 (20), 80 (100). Anal. calcd for C_14_H_12_N_4_O_2_S (300.07): C, 55.99; H, 4.03; N, 18.66; S, 10.67. Found: C, 55.85; H, 3.91; N, 18.59; S, 10.80.

*1-(4-Methyl-2-phenylthiazole-5-carbonyl)-1,2-dihydropyridazine-3,6-dione* (**21**). White crystals (83%); mp = 112–114 °C (EtOH); IR (KBr) *v* = 3228 (NH), 1693, 1677 (3C=O) cm^−1^; ^1^H-NMR (DMSO-*d_6_*, 300 MHz): *δ* = 2.63 (3H, s, thiazole–CH_3_), 6.42 (1H, d, CH=), 6.84 (1H, d, CH=), 7.53-8.03 (5H, m, Ar–H), 11.36 (1H, s, NH); ^13^C-NMR (DMSO-*d_6_*, 75 MHz): *δ* = 17.9 (CH_3_-thiazole), 127.8, 128.8, 129.8, 130.5, 131.1, 132.6, 133.6, 143.9, 146.9, 158.9, 161.8, 167.6. EIMS *m*/*z* (%): 313 [M^+^] (80), 202 (100), 71 (50). Anal. calcd for C_15_H_11_N_3_O_3_S (313.05): C, 57.50; H, 3.54; N, 13.41; S, 10.23. Found: C, 57.63; H, 3.45; N, 13.29; S, 10.32.

*2-(4-Methyl-2-phenylthiazole-5-carbonyl)-2,3-dihydrophthalazine-1,4-dione* (**23**). Yellowish-white crystals (84%); mp = 270–272 °C (AcOH); IR (KBr) *v* = 3231 (NH), 1691, 1671 (3C=O) cm^−1^; ^1^H-NMR (DMSO-*d_6_*, 300 MHz): *δ* = 2.70 (3H, s, thiazole–CH_3_), 7.55-8.04 (9H, m, Ar–H), 11.22 (1H, s, NH); ^13^C-NMR (DMSO-*d_6_*, 75 MHz): *δ* = 18.2 (CH_3_-thiazole), 125.9, 126.8, 127.4, 128.8, 129.8, 130.4, 131.3, 132.5, 133.7, 142.9, 143.7, 145.9, 158.9, 160.8, 161.8, 167.6. EIMS *m*/*z* (%): 363 [M^+^] (50), 202 (100), 71 (40). Anal. calcd for C_19_H_13_N_3_O_3_S (363.07): C, 62.80; H, 3.61; N, 11.56; S, 8.82. Found: C, 62.63; H, 3.52; N, 11.49; S, 8.73.

### 3.2. The In Silico Studies

#### 3.2.1. Molecular Dynamics Simulation

Nanoscale Molecular Dynamics (NAMD) and Visualizing Molecular Dynamics (VMD) software were used in the molecular dynamics study (MDS) using the standard CHARMM 36 forcefield, and the proteins were solvated with the TIP3P water model [44,45,46,47]. The temperature of 310 K and the 154 mM NaCl concentration were adjusted during the simulation period to simulate the protein at its physiological conditions. The MDS calculations were done on the Bibliotheca Alexandrina supercomputer, Alexandria, Egypt. Chimera software was used to cluster the trajectories after MDS using default parameters [48]. Five representative structures of SARS-CoV-2 M^pro^ were used in the docking study after clustering the trajectories of the MDS of the M^pro^ (PDB ID: 6Y84).

#### 3.2.2. Molecular Docking

AutoDock Vina 1.1.2 software was used to test the thiazole compounds against SARS-CoV-2 main protease different conformations, while Nelfinavir was used as a positive control. The ligands were prepared using the PyMOL 2.4 and AutoDock Tools 1.5.6 software [49]. Any missed hydrogen atoms were added, followed by charges addition before input files generation. The docking utilizes flexible active site (H41 and C145) and flexible ligand protocol. The AutoDock Vina grid box size used in the docking experiments is 40 × 40 × 40 Å^3^, while the box centers were set to be at the dyads H41 and C145 (−3.9, 19.7, −5.0) Å, (−4.6, 18.9, −15.5) Å, (−8.2, 37.0, −8.4) Å, (4.7, 38.8, −3.3) Å, and (22.1, 32.0, −2.4) Å, for the different conformations of the SARS-CoV-2 M^pro^. Docking calculations were performed on a local workstation (Intel core i7/12 GB RAM). After docking, the complexes were investigated using both PyMOL and the Protein–Ligand Interaction Profiler (PLIP) 2.1.4 software [42].

## 4. Conclusions

In this context, a series of novel hydrazones (**3a**–**c**, **9**, **11a**–**c**), pyarzoles (**13**, **15**, **17**, **19**), and pyrazines (**21**, **23**) were synthesized using a solvent-drop, eco-friendly, environmental grinding method. The structure of the newly prepared compounds was established based on both elemental analysis and spectroscopic data and by an alternative method wherever possible. Also, the anti-viral activity of all the products was tested against SARS-CoV-2 main protease (M^pro^) using molecular docking combined with molecular dynamics simulation (MDS). The average binding affinities of the compounds **3a**, **3b**, and **3c** were better than that of the positive control Nelfinavir. So, these compounds can bind effectively to SARS-CoV-2 M^pro^ and hence, contradict the virus lifecycle.

## Figures and Tables

**Table 1:** The interaction pattern of the thiazole compounds (**3a**, **3b**, **3c**, **9**, **11a**, **11b**, **11c**, **13**, **15**, **17**, **19**, **21**, and **23**) and Nelfinavir against SARS-CoV-2 M^pro^. The AutoDock Vina scores are listed among the number of H-bonds and hydrophobic contacts and the residues that interact. Bold residues are the most interacting residue E166.

Compound	AutoDock Vina Score	H-Bonding		Hydrophobic Interaction	
		Number	Residues from SARS-CoV-2 M^pro^ Take Part in the Interaction	Number	Residues from SARS-CoV-2 M^pro^ Take Part in the Interaction
**Nelfinavir**	−6.7	1	**E166**	3	T25, L27, and M165
**3a**	−8.1	7	N142, G143, S144(2), **E166**, D187, Q189	1	**E166**
**3b**	−8.3	7	N142, G143, S144(2), **E166**, D187, Q189	1	**E166**
**3c**	−8.3	7	L141, N142, G143, S144, **E166**, D187, Q189	1	**E166**
**9**	−7.3	1	D187	3	T25, L27, Q189
**11a**	−6.6	1	**E166**	4	L27, N142, M165, **E166**
**11b**	−6.7	1	**E166**	3	T25, L27, **E166**
**11c**	−6.4	1	G143	2	L27, **E166**
**13**	−6.4	1	Q189	0	N/A
**15**	−6.9	3	G143, S144, C145	2	**E166**, Q189
**17**	−7.0	0	N/A	2	T25, Q189
**19**	−7.0	6	N142, G143, S144, C145, **E166**, Q189	1	Q189
**21**	−7.0	3	G143, S144, **E166**	4	T25, L27(2), **E166**
**23**	−7.3	3	G143, S144, C145	1	**E166**

N/A: Not available.

## Supplementary Materials

The following are available online, Figure S1: ^1^H-NMR, ^13^C-NMR and Mass spectra of compound **3a**, Figure S2: ^1^H-NMR, ^13^C-NMR and IR spectra of compound **9**, Figure S3: ^1^H-NMR and IR spectra of compound **15**, Figure S4: ^1^H-NMR and IR spectra of compound **23**, Figure S5: Molecular Simulations, Figure S6: Molecular Simulations (RMSF).
