# Supplementary material for: Clean Grinding Technique: A Facile Synthesis and In Silico Antiviral Activity of Hydrazones, Pyrazoles, and Pyrazines Bearing Thiazole Moiety against SARS-CoV-2 Main Protease (Mpro)
Source: Molecules. 2020 Oct 6;25(19):4565. doi: 10.3390/molecules25194565 (PMC7582706; doi:10.3390/molecules25194565)
Supplement: Supplementary file 1 [file molecules-25-04565-s001.pdf]

Clean Grinding Technique: A Facile Synthesis and In silico Antiviral Activity of Hydrazones, Pyrazoles, and Pyrazines Bearing Thiazole Moiety Against SARS-CoV-2 main protease (M^{pro})

Sraa Abu-Melha [1], Mastoura M. Edrees [1,2]*, Sayed M. Riyadh [3,4], Mohamad R. Abdelaziz [5], Abdo A. Elfiky [6], Sobhi M. Gomha [3,7]*

[1] Department of Chemistry, Faculty of Science, King Khalid University, Abha 61413, SA

[2] Department of Organic Chemistry, National Organization for Drug Control and Research (NODCAR), Giza 12311, Egypt

[3] Department of Chemistry, Faculty of Science, Cairo University, Giza, 12613, Egypt

[4] Department of Chemistry, Faculty of Science, Taibah University, Al-Madinah Al-Munawarah, 30002, Saudi Arabia

[5] Department of Pharmaceutical Chemistry, Faculty of Pharmacy, MIU University, Egypt

[6] Biophysics Department, Faculty of Science, Cairo University, Giza, Egypt

[7] Department of Chemistry, Faculty of Science, Islamic University in Al-Madinah Al-Munawarah, 42351, Saudi Arabia

* Corresponding authors: s.m.gomha@gmail.com, mstorh@kku.edu.sa, Tel.: +20-237-400-304, +966545888764; Fax: +20-025-685-799

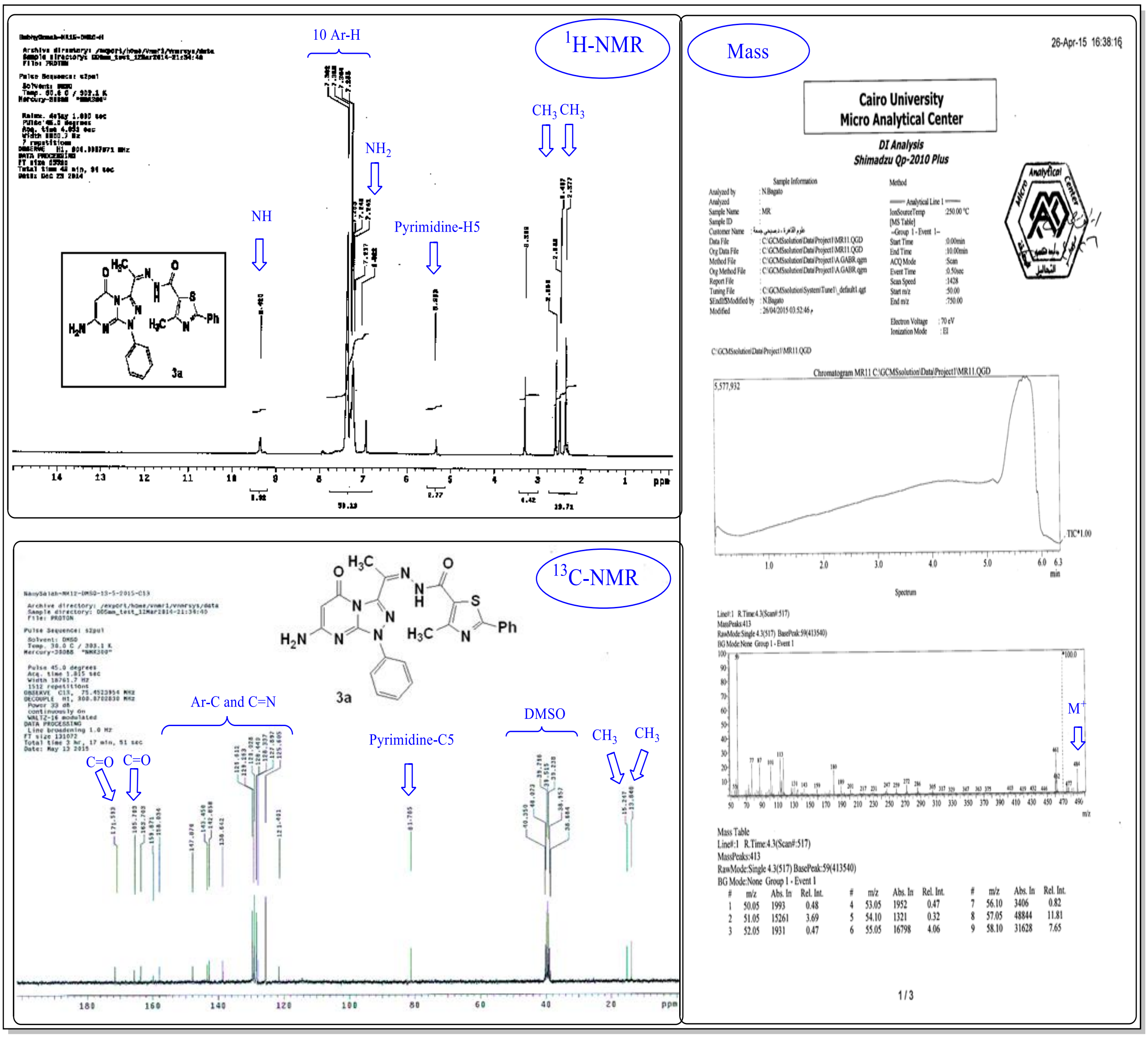

Figure S1. ^{1}H-NMR, ^{13}C-NMR and Mass spectra of compound **3a.**

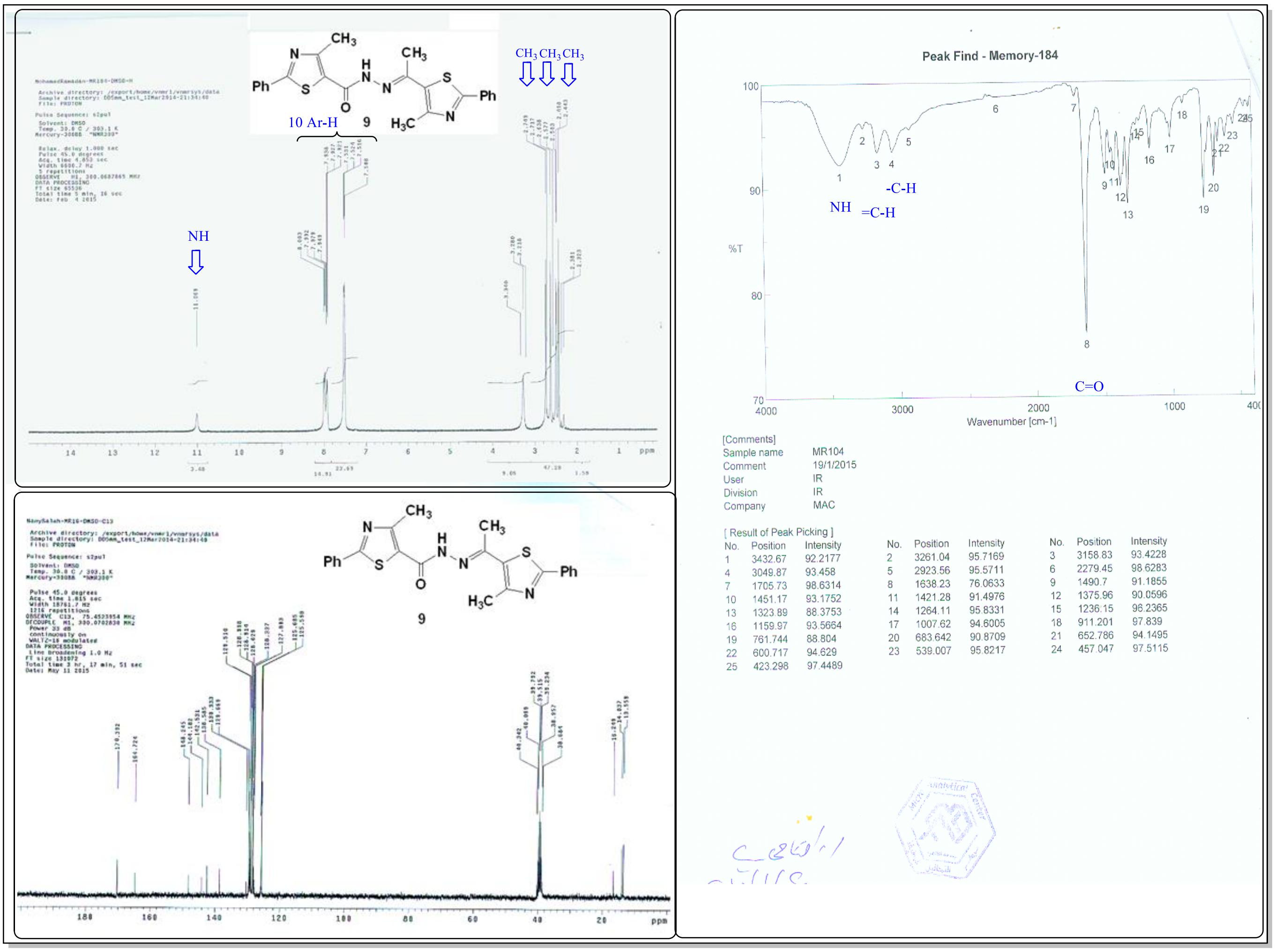

Figure S2. ^{1}H-NMR, ^{13}C-NMR and IR spectra of compound **9.**

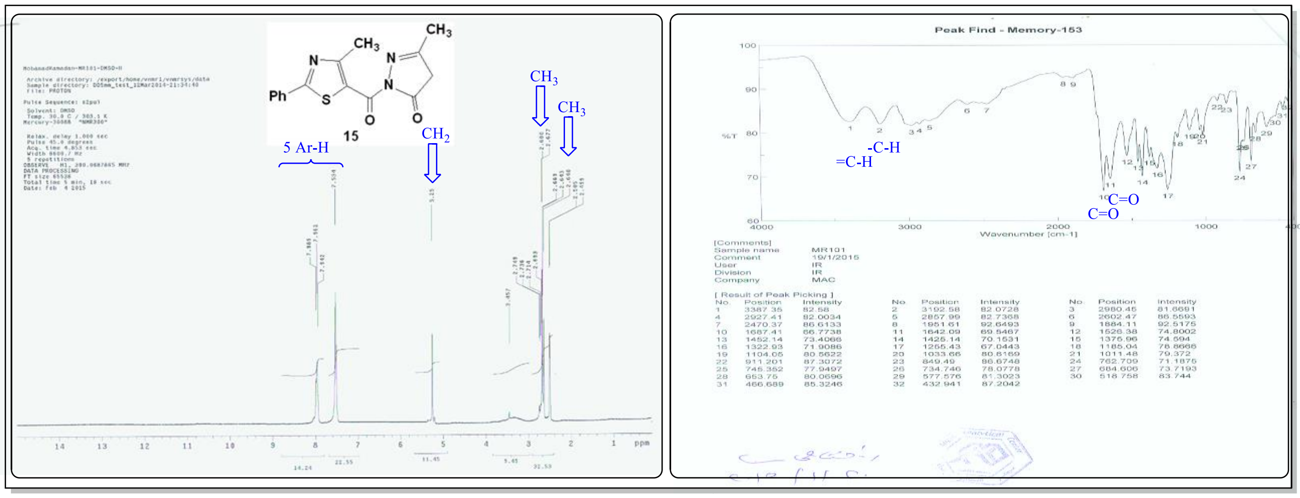

Figure S3. ^{1}HNMR and IR spectra of compound **15.**

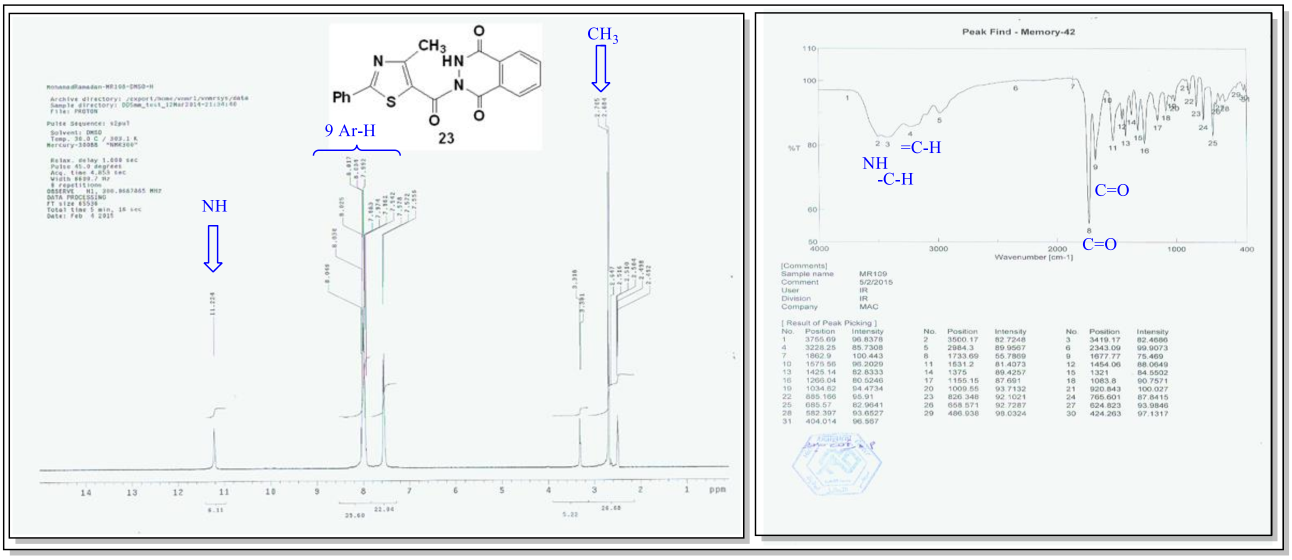

Figure S4. ^{1}HNMR and IR spectra of compound **23.**

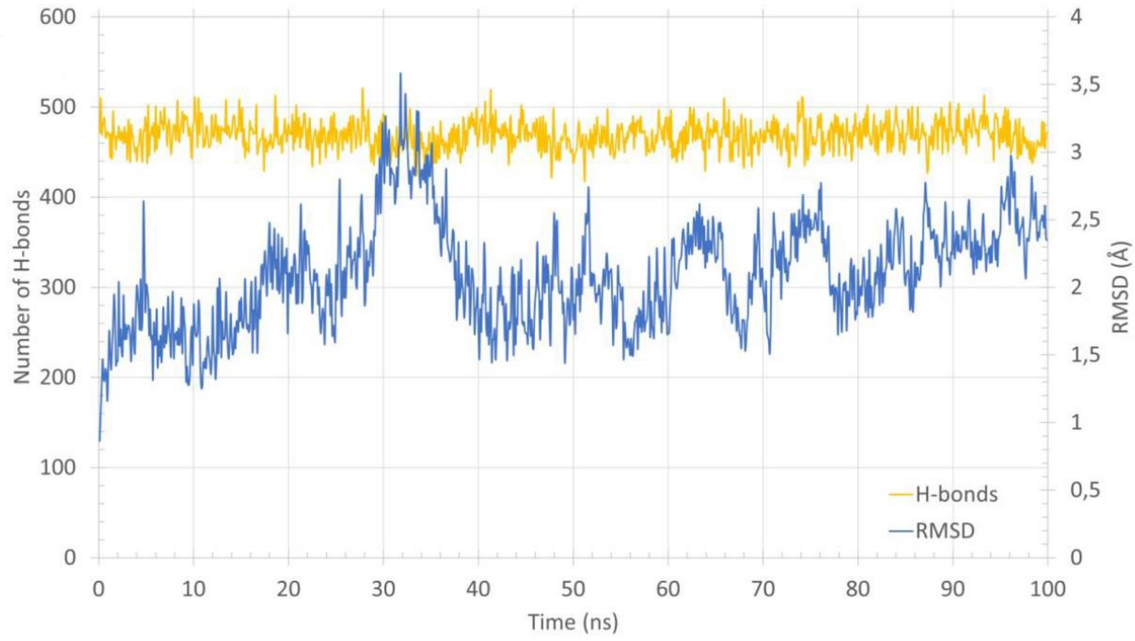

Figure S5. Molecular Simulations.

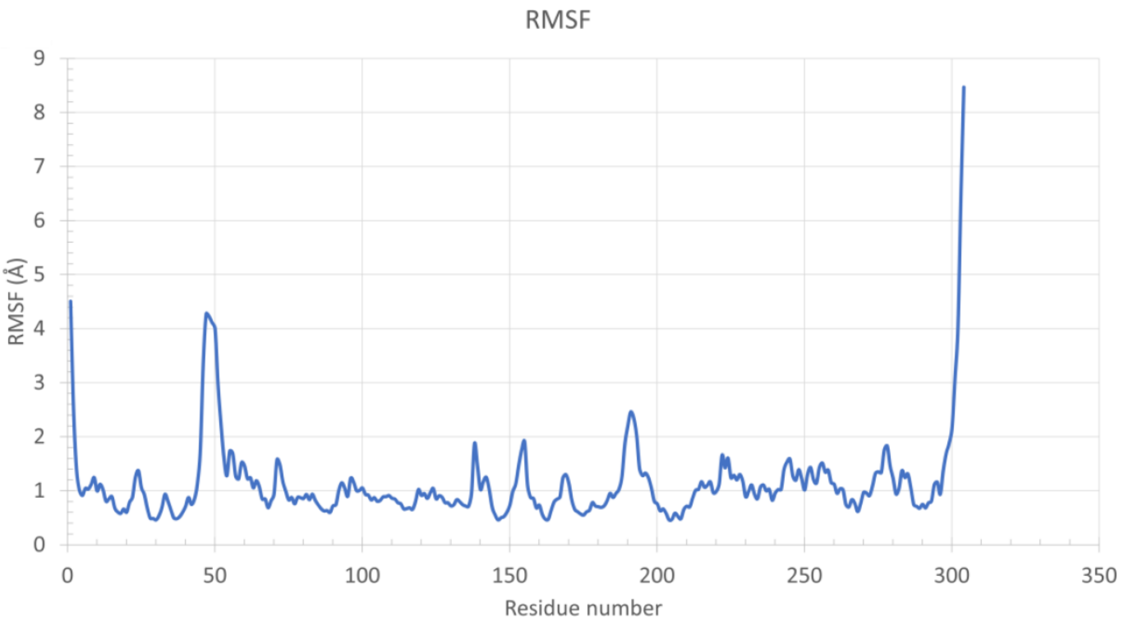

Figure S6. Molecular Simulations (RMSF).
